# Management of HR+/HER2+ lobular breast cancer and trends do not mirror better outcomes

**DOI:** 10.1016/j.breast.2022.05.005

**Published:** 2022-05-25

**Authors:** Marita Yaghi, Nadeem Bilani, Barbara Dominguez, Iktej Singh Jabbal, Carlos Rivera, Maroun Bou Zerdan, Hong Li, Diana Saravia, Elizabeth Stone, Zeina Nahleh

**Affiliations:** a Department of Hematology-Oncology, Maroone Cancer Center, Cleveland Clinic Florida, Weston, FL, USA; b Department of Medicine, New York-Presbyterian Brooklyn Methodist Hospital, New York, NY, USA; c Department of Internal Medicine, SUNY Upstate Medical University, Syracuse, NY, USA; d Department of Quantitative Health Sciences, Cleveland Clinic Foundation, Cleveland Clinic, OH, USA

**Keywords:** Breast Neoplasms, Carcinoma, Lobular, Antineoplastic protocols

## Abstract

**Purpose:**

Treatment protocols for invasive lobular breast cancer (ILC) have largely followed those for invasive ductal breast cancer. This study compares treatment outcomes of endocrine therapy versus combined chemo-endocrine therapy in hormone-receptor-positive (HR+), HER2-positive (HER2+) ILC tumors in a large national registry.

**Methods:**

We sampled the National Cancer Database (2010–2016) for female patients with stages I-III, HR+/HER2+ ILC who underwent surgery. Cochran-Armitage trend test examined trends of treatment regimen administration: Surgery only (S), chemotherapy (C), endocrine therapy (ET), and combined chemo-endocrine therapy (CET), with or without anti-HER2 therapy. Cox proportional hazard model were used to compare overall survival (OS) across ET and CET cohorts, stratifying for anti-HER2 therapy, before and after propensity score match of cohorts (2013–2016). Kaplan-Meier (KM) survival curves were also produced.

**Results:**

*N*=11,421 were included. 58.7% of patients received Anti-Her2 therapy after 2013. CET conferred better OS over ET in the unmatched (adjusted-5-year-OS: 92.5% vs. 81.1%, ***p<0.001***) and PS-matched (90.4% vs. 84.5%, ***p=0.001***) samples. ET caused lower OS in patients who received Anti-Her2 therapy (HR: 2.56, 95% CI: 1.60–4.12, ***p<0.001***) and patients who did not (HR: 1.84, 95% CI: 1.21–2.78, ***p=0.004***), as compared to CET on multivariable analysis. KM modeling showed highest OS in the CET cohort who received Anti-Her2 (93.0%), followed by the CET cohort who did not receive Anti-Her2 (90.2%) (***p=0.06***).

**Conclusion:**

Chemotherapy followed by endocrine therapy and Anti-Her2 therapy was shown to be the most effective treatment modality in HR+/HER2+ ILC, contrasting previous data on the inconclusive benefit of chemotherapy in patients with ILC.

## Highlights

• Less chemotherapy (CT) is used in favor of endocrine therapy (ET) alone for HR+/HER2+ ILBC at the expense of efficacy.
• CT followed by ET remains an effective treatment in HR+/HER2+ ILBC, specifically for larger and locally advanced tumors.
• Research investigating subgroups of lobular BC who would benefit from CT de-escalation, or ET alone without CT is needed.

## 1 Introduction

Invasive lobular breast cancer (ILC) accounts for approximately 15% of all invasive breast cancers (BC) [1], and is the second most common type of breast cancer after invasive ductal carcinoma (IDC). Historically considered a homogenous tumor type, recent findings highlight both the heterogeneity within ILC and its differences with IDC. The association between ILC and advanced age, advanced pathologic T stage, higher likelihood of lymph node invasion with increasing tumor size [2], and lower histologic grade, as compared to IDC is now well-established [3,4]. Clinically, ILC tumors have an indolent clinical course and grossly manifest as ill-defined masses or architectural distortion [5]. Additionally, ILC is generally more difficult to detect through standard imaging modalities such as mammography, Fluorodeoxyglucose-Positron Emission Tomography (FDG-PET), or ultrasounds compared to IDC [[6], [7], [8]].

While the hallmark pathological characteristic of ILC remains the loss of E-cadherin (CDH1) protein expression [7], the unique genomic profile of these tumors also harbors mutations in various proteins related to impaired cell adhesion, such as α-catenin/CTNNA1 [9]. Consequently discohesive tumor cell growth in the stroma in a single-file pattern [10] is referred to as the “classical subtype”, but other subtypes have also been described based on tumor architecture (alveolar, solid, and trabecular) or cell cytonuclear characteristics (apocrine, histiocytoid, pleomorphic, and signet ring) [11]. These are further grouped as “mixed/non-classical subtype”, and have been associated with worse survival as compared to the classical one [12]. Additionally, these tumors have a genomic profile characterized by a higher prevalence of mutations in serine/threonine-protein kinase *AKT1*, and *HER2* and *HER3*, two members of the human epidermal growth factor receptor (HER), compared to IDC [11], resulting in increased activation of the human epidermal growth factor receptor pathway. However, the clinical differences between ILC subtypes remain largely understudied.

Despite ILC's distinct clinical features, treatment protocols have largely mirrored those for IDC [13]. Large trials investigating the benefit of chemotherapy in endocrine-sensitive BC tumors have not looked at ILC as a separate subtype [14]. Moreover, recent data highlights the heterogeneity within ILC, but a paucity of large-scale studies focusing on subgroups of ILC has led to inconclusive results on the benefit of chemotherapy in ILC in general as well as in each particular subtype [1,15].

This study aims at assessing the benefit of chemotherapy and Anti-Her2 targeted therapy in the hormone receptor positive (HR+) – estrogen positive (ER+) and/or progesterone positive (PR+) – *and* HER2 positive (HER2+) subtype of ILC, and evaluating *anti-HER2 targeted therapy*. Patterns of use over time and the benefit of endocrine therapy alone versus chemotherapy followed by endocrine therapy will be evaluated using the National Cancer Database (NCDB).

## 2 Methods

### 2.1 Patient data

The NCDB is a national registry jointly supported by the Commission on Cancer (CoC) and the American College of Surgeons (ACS), which gathers information from over 1500 medical institutions and includes over 70% of all cancer patients in the United States [16].

**Fig. 1:**
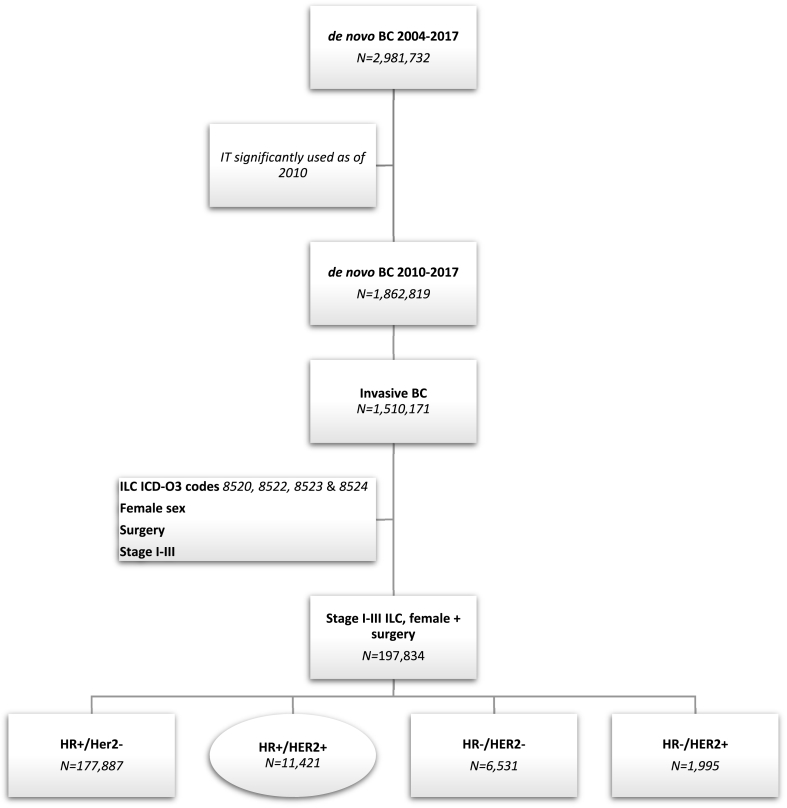
Case selection diagram.

We sampled the NCDB (2010–2016) for female patients diagnosed with ILC, AJCC stages I-III and with HR+ and HER2+ receptor subtype, using the International Classification of Diseases (ICD) diagnostic codes. HR and HER2 status were determined using site-specific variables within the database. ER and PR assays were used to determine HR status using a 1% cell stain cutoff for positivity. A combination of Immunohistochemistry (IHC), Fluorescent In Situ Hybridization (FISH), Chromogenic In Situ Hybridization (CISH) testing were used to determine HER2 amplification/overexpression. Only patients who underwent surgery were included. Fig. 1 outlines the selection process used. Patients were divided into 4 distinct cohorts based on treatment regimen administration: **1.** Surgery only (S), **2.** Chemotherapy (CT), **3.** Endocrine therapy (ET), and **4.** Combined Chemotherapy followed by Endocrine therapy (CET).

Information pertaining to patient age, sex, gender, insurance status, morbidity as reflected by Charslon/Deyo score, tumor site, stage, grade, ILC histology, OncotypeDX (ODX) radiation therapy, Anti-Her2 therapy and overall survival (OS) were collected. To note, endocrine reflects the use of Aromatase Inhibitors and Anti-Her2 therapy reflects the use of trastuzumab, as per reporting guidelines to the registry.

Patients who were treated with Surgery only or Chemotherapy only accounted for roughly 15.3% of all patients. They were excluded consequently excluded from the remainder of the analysis to eliminate confounding factors.

All statistical analyses were performed using SAS version 9.4. Two-sided p-values were computed with *p*<0.01 was considered as statistically significant due to the large sample size.

### 2.2 Analyzing treatment trends in HR+/HER2+ ILC

We used the Cochrane-Armitage test to explore the trends of treatment administration in our population from 2010 to 2016. We then further stratified our population according to the usage of anti-Her 2 therapy. As the use of Anti-Her2 therapy formally began around 2013, we used the Cochrane-Armitage test to look at the trends of treatment regimen administration between the years 2013–2016. Bivariate analysis with chi-squared and *t*-test statistics was then performed to compare patient sociodemographic and tumor clinicopathologic characteristics in relation to targeted anti-Her2 therapy administration in the overall patient population, and in the ET and CET cohorts.

### 2.3 Survival analysis

The Kaplan-Meier (KM) method was used to compare the 3-year and 5-year OS across all 4 patient cohorts before (2010–2013) and after (2013–2016) the start of Anti-Her2 therapy administration.

Focusing on the years 2013–2016, OS was also analyzed stratifying for Anti-Her2 therapy administration. A multivariable Cox proportional hazard model (significance p < 0.005) was then used to identify factors impacting patient OS across the 2 main treatment cohorts, ET and CET, stratifying for the use of anti her2 therapy. It was followed by Propensity Score (PS) matching with 0.1 caliper in logistic regression model to identify patients with comparable demographics and clinical characteristics across the ET and CET treatment groups. The model included age, lobular histology, stage, comorbidity score, radiation therapy and insurance type, with exact match of AJCC stage. KM survival curves were estimated separately for participants in the ET and CET cohorts in both the unmatched sample and the propensity score matched sample. 3-year and 5-year OS was then estimated in both the unmatched sample and matched pairs in the ET and CET treatment cohorts.

KM survival were also estimated for participants in the overall ET and CET cohorts from the unmatched sample, stratifying for the use of Anti-Her2.

## 3 Results

### 3.1 Patient data and trends in treatment utilization

A total of ***N***=11,421 female patients diagnosed with HR+ (ER+ and/or PR+) and Her2+ stage I-III ILC between 2010 and 2016 and received surgery were identified from the NCDB. They represented 5.8% of all ILC patients treated with surgery from the NCDB. Patient selection process was summarized in Fig. 1. Overall, 819 (7.2%) patients received Surgery only, 928 (8.1%) received CT, 2134 (18.7%) received ET and 7540 (66.0%) received CET.

**Fig. 2:**
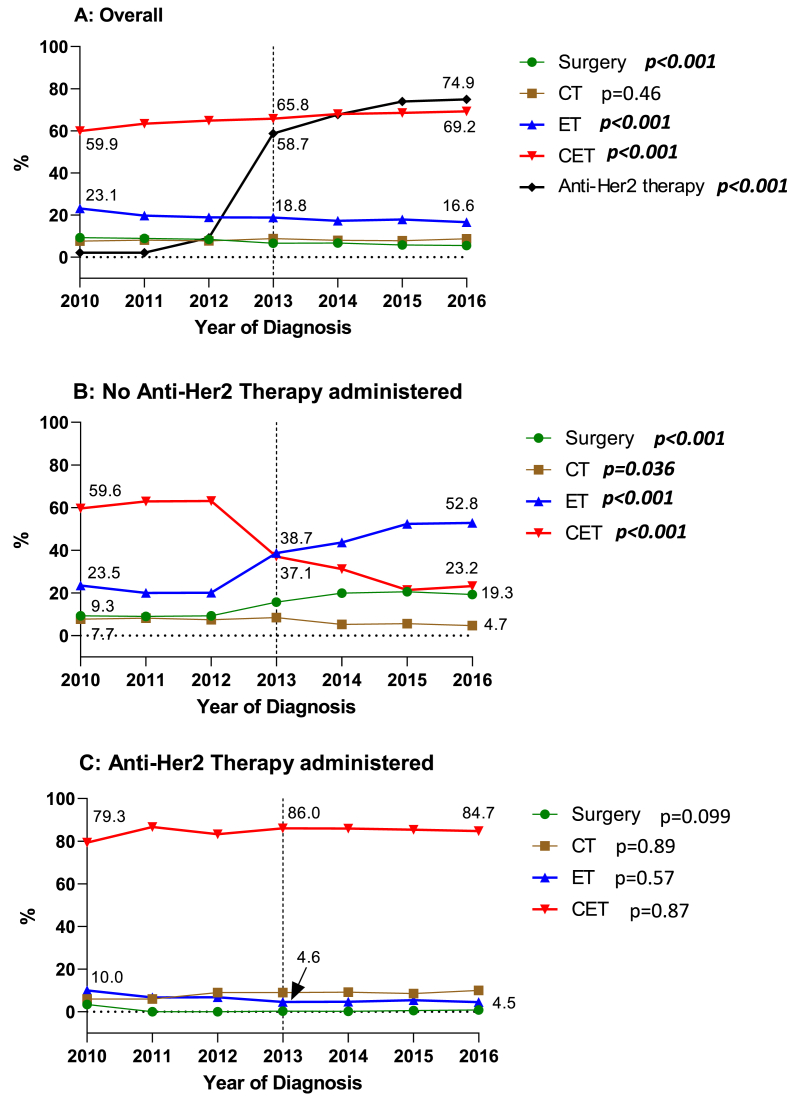
Treatment trends in stage I-III HR+/HER2+ ILC over time.

Over time, the number of patients treated with surgery only decreased from 9.3% in 2010 to 5.5% in 2016 (***p<0.001***) while the proportion of patients treated with CT did not significantly change (p = 0.46). The use of ET significantly decreased from 23.1% in 2010 to 16.6% in 2016 (***p<0.001***), while the use of CET significantly increased from 59.9% in 2010 to 69.2% in 2016 (***p<0.001***). A change in the utilization pattern of Anti-Her2 therapy can be seen in 2013 with 58.7% of patients receiving Anti-Her2 therapy as compared to 2.1–9.2% in 2010–2012; the numbers continue to significantly increase to reach 74.9% in 2016 (***p<0.001***) (Fig. 2A).

Stratifying for Anti-Her2 therapy, trends in systemic treatment utilization shifted between 2013 and 2016. In the strata of patients who did not receive Anti-Her2 therapy (N = 1646), a net increase in ET utilization from 38.7% in 2013 to 52.8% in 2016 was observed (***p<0.001***), coupled with a significant decrease in CET use from 37.1% in 2013 to 23.2% in 2016 (***p<0.001***) (Fig. 2B). In contrast, in the strata who received Anti-Her2 therapy (N = 4946), the use of all treatments remains steady over time. Focusing on ET and CET, 4.6% of patients received ET in 2013 as compared to 4.5% in 2016 (p=0.57) and 86.0% of patients received CET in 2013 as compared to 84.7% in 2016 (p=0.87) (Fig. 2C).

### 3.2 Predictors of Anti-Her2 therapy administration

**Fig. 3:**
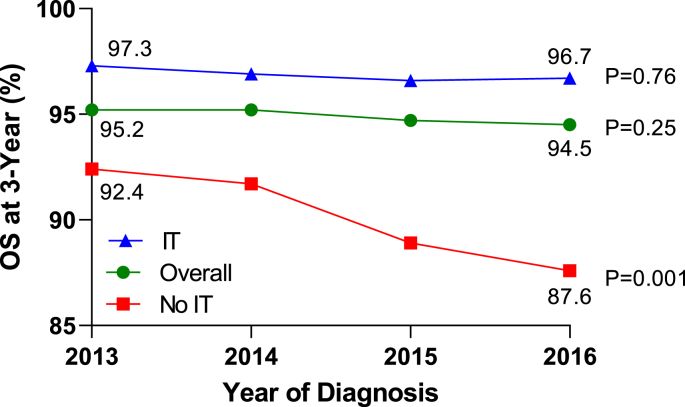
OS after the introduction of Anti-Her2 therapy, stratifying for the use of Anti-Her2 therapy.

The use of Anti-Her2 therapy had a clear survival benefit in the patients treated with either ET or CET between 2013 and 2016: 3-year OS in the Anti-Her2 strata was superior to OS in the no Anti-Her 2 strata (Fig. 3).

Analysis of the clinicopathologic characteristics of patients in both the ET and CET treatment cohorts (N=6110) (2013–2016) reveals that most patients (60.1%) were under the age of 64, had no comorbidities as per the Charlson/Deyo score (83.7%) and had private insurance (54.6%). Stage I tumors (47.1%) were most prevalent in that patient population, followed by stage II (38.8%) and then stage III (14.1%). Mixed lobular histology (60.8%) was more prevalent than classical histology. On treatment administration, RT was administered to 63.0% of patients and more patients received CET (79.5%) that ET (Table 1).

**Table 1:** Clinicopathologic characteristics of patients with HR+/HER2+ stages I-III ILC (2013–2016) and bivariable association of characteristics A) with Anti-Her2 therapy administration in the overall Sample B) with CET and ET administration in the Anti-Her2 therapy strata C) with CET and ET administration in the No Anti-Her2 therapy strata.

Factor	Total *N* = *6110*				Anti-Her2 therapy strata *N* = *4464*			No Anti-Her2 therapy strata *N* = *1646*		
	Overall	Anti-Her2 therapy	No Anti-Her2 therapy	Chi-square p-value	ET cohort	CET cohort	Chi-square p-value	ET cohort	CET cohort	Chi-square p-value
**Patient number *N (%)***		4464(73.1)	1646(26.9)	***<0.001***	239(5.4%)	4225(94.6%)		1011(61.4)	635(38.6)	
**CET administration**	4860(79.5)	4225(86.9)	635(13.1)	***<0.001***	–	–		–	–	
**Age (mean ± SD)**	61.1 ± 13.0	58.8 ± 12.1	65.6 ± 13.3	***<0.001***	71.9 ± 12.0	57.9 ± 11.6	***<0.001***	69.4 ± 12.5	59.4 ± 12.1	***<0.001***
**Age category**				***<0.001***			***<0.001***			***<0.001***
<55	1982(31.6)	1627(82.1)	355(17.9)		25(10.9)	1602(37.9)		135(13.4)	220(34.6)	
55–64	1740(28.5)	1354(77.8)	386(22.2)		27(11.3)	1327(31.4)		206(20.4)	180(28.3)	
65–74	1886(30.9)	1118(59.3)	471(40.7)		78(32.6)	1040(24.6)		296(29.3)	175(27.6)	
≥75	798(13.1)	364(45.6)	434(54.4)		108(45.2)	256(6.1)		374(37.0)	60(9.4)	
**Charlson/Deyo Score of 0**	5116(83.7)	3813(75.5)	1303(24.5)	***<0.001***	182(76.2)	3631(85.9)	***<0.001***	775(76.7)	528(83.1)	***0.002***
**Private Insurance status**	3339(54.6)	2612(78.2)	727(21.8)	***<0.001***	61(25.5)	2551(60.4)	***<0.001***	367(36.8)	360(57.2)	***<0.001***
Tumor **stage**				***<0.001***			0.14			***<0.001***
**Stage I**	2879(43.7)	1928(67.0)	951(33.0)		124(51.9)	1804(42.7)		696(68.8)	255(40.2)	
**Stage II**	2368(35.9)	1847(78.0)	521(22.0)		80(33.5)	1767(41.8)		266(26.3)	255(40.2)	
**Stage III**	862(13.1)	688(79.8)	174(20.2)		34(14.6)	654(15.5)		49(4.8)	125(19.7)	
**ILC mixed histology**	3715(60.8)	2784(75.0)	931(25.0)	***<0.001***	116(48.5)	2668(63.1)	***<0.001***	558(55.2)	373(58.7)	0.16
**RT administration**	3852(58.4)	2965(77.0)	887(23.0)	***<0.001***	122(51.0)	2843(67.3)	***<0.001***	492(48.8)	395(62.6)	***<0.001***

Comparing Anti-Her2 therapy usage across the ET and CET treatment cohorts (N = 6110) (2013–2016), bivariate analysis reveals a significant difference in Anti-Her2 therapy administration (***p<0.001***): 73.1% of patients received Anti-Her2 therapy while 26.9% did not (***p<0.001***). Subsequent bi-variable analysis of patient sociodemographic and tumor clinicopathologic characteristics reveal that the following factors influence the likelihood of Anti-Her2 therapy administration: **1) CET administration**, 86.9% of patients in the CET cohort received Anti-Her2 therapy as well while 13.1% did not (***p<0.001***). **2) Age**, patients who received Anti-Her2 therapy were more likely to be younger, with an average age of 58.8 ± 12.1 as compared to 65.6 ± 13.3 (***p<0.001***). Specifically, patients under the age of 74 were more likely to receive Anti-Her2 therapy (***p<0.001***). **3) Charlson/Deyo score**, patients who were otherwise healthy were more likely to receive Anti-Her2 therapy (75.5%) (***p<0.001***). **4) Insurance status**, patients on a private insurance plan (78.2%) were more likely to receive Anti-Her2 therapy (***p<0.001***). **5) AJCC stage**, tumors with stage II (78.0%) or stage III (79.8%) were more likely to be treated with Anti-Her2 therapy than stage I tumors (67.0%) (***p<0.001***). **6) Radiation therapy**, patients who received radiation were more likely to also receive Anti-Her2 therapy (77.0%) (***p<0.001***) (Table 1).

Focusing on the strata of patients that received Anti-Her2 (N = 4946), significant patient and tumor characteristics differed between the ET and CET cohorts. Overall, patients were more likely to receive CET (94.6%) than ET (5.4%) (***p<0.001***). Patients in the ET cohort were older with a mean age of 71.9 ± 12.0 and compared to 57.9 ± 11.6 in the CET cohort (***p<0.001***). Those is the CET cohort were more likely to be younger than 64 years of age: 37.9% aged <55 and 31.4% between 55 and 64, while those in the ET cohort were more likely to be above 65 years of age: 32.6% aged between 65 and 74 and 45.2% aged ≥75 (***p<0.001***). Those in the CET cohort were more likely to have no comorbidities (85.9%) and have private insurance (60.4%) as compared to those in the ET cohort (76.2% and 25.5% respectively) (***p<0.001***). Tumors from the CET cohort were more likely to harbor mixed histology (63.1%) and be treated with RT (67.3%) as compared to those in the ET cohort (48.5% and 51.0% respectively) (***p<0.001***). Stage distribution was uniform across both cohorts (p=0.14) (Table 1).

In the strata of patients who did not receive Anti-Her2 therapy (N = 1646), patients were more likely to receive ET (61.4%) than CET (38.6%). Mean patient age was higher in the ET cohort (69.4 ± 12.5) than in the CET cohort (59.4 ± 12.1) (***p<0.001***). Patients in the ET cohort were also more likely to be older than 65 years of age: 29.3% were aged 65–74 and 37.0% were aged ≥75 as compared to 27.6% and 9.4% in the CET cohort respectively. Those in the CET cohort were more likely to have 0 comorbidities (83.1% vs. 76.7%) (***p=0.002***) and have private insurance (57.2% vs. 36.8%) (***p<0.001***). Stage I tumors were more common in the ET group (68.8% vs. 40.2%) while stage II (40.2%) and stage III (19.7%) were more common in the CET group as compared to ET (26.3% and 4.8% respectively) (***p<0.001***). RT administration was more common in the CET cohort (62.6%) than the ET cohort (48.8%) (***p<0.001***). Tumor histology was uniform across both cohorts (p = 0.16) (Table 1).

### 3.3 Propensity-scoring and matched pairs survival outcomes

A total of N = 6110 patients diagnosed between 2013 and 2016 received either ET (n = 1,250, 20.5%) or CET (n = 4,860, 79.5%). Significant differences existed between the 2 cohorts for patient and tumor characteristics, which were corrected by the matching process. The patient in the CET cohort were more likely to have stage II (41.6% vs. 27.7%) or stage III disease (16.0% vs. 6.6%) (***p<0.001***), be younger (average age 58.1 vs. 69.9) (***p<0.001***), have no comorbidities (85.6% vs. 77.0%) (***p<0.001***), have ILC tumors with mixed histology (62.6% vs. 53.9%) (***p<0.001***), be privately insured (59.9% vs. 34.2%), receive radiation therapy (66.6% vs. 49.1%) and receive Anti-Her2 therapy (89.9% vs. 19.1%) (***p<0.001***). Propensity score match of the CET and ET cohorts yielded a total of 661 pairs (N = 1332) with exact stage match: 379 pairs (57.3%) with stage I, 207 pairs (31.3%) with stage II and 75 pairs (11.3%) with stage III (p = 0.99). Within each stratum of the propensity score, age, Charlson/Deyo score, ILC histology, private insurance status, radiation therapy administration and Anti-Her2 therapy administration had similar means or prevalence with reductions in standardized differences to less than 5% (Table 2).

**Table 2:** Clinical characteristics of patients in the ET and CET cohorts before and after PS match (2013–2016).

Variable	Before PS-match				After PS match			
	Total	ET	CET	p-value	Total	ET	CET	p-value
	(N = 6110)	(N = 1250)	(N = 4860)		(N = 1322)	(N = 661)	(N = 661)	
Tumor **stage**				***<0.001***				0.99
Stage I	2879(43.7)	820(65.7)	2059(42.3)		758(57.3)	379(57.3)	379(57.3)	
Stage II	2368(35.9)	346(27.7)	2022(41.6)		414(31.3)	207(31.3)	207(31.3)	
Stage III	862(13.1)	83(6.6)	779(16.0)		150(11.3)	75(11.3)	75(11.3)	
**Age**	61.1 ± 13.0	69.9 ± 12.4	58.1 ± 11.7	***<0.001***	65.4 ± 12.2	65.6 ± 12.7	65.2 ± 11.7	0.51
**Charslon/Deyo score of 0**	5116(83.7)	962(77.0)	4159(85.6)	***<0.001***	1073(81.2)	535(80.9)	538(81.4)	0.83
**ILC mixed histology**	3715(60.8)	674(53.9)	3041(62.6)	***<0.001***	733(55.4)	363(54.9)	370(56.0)	0.70
**Private insurance**	3339(54.6)	428(34.2)	2911(59.9)	***<0.001***	572(43.3)	290(43.9)	282(42.7)	0.66
**Radiation therapy**	3852(28.4)	614(49.1)	3228(66.6)	***<0.001***	729(55.1)	354(53.6)	375(56.7)	0.25
**Anti-Her2 therapy administration**	4464(73.1)	239(19.1)	4225(86.9)	***<0.001***	467(35.3)	230(34.8)	237(35.9)	0.69

**Fig. 4:**
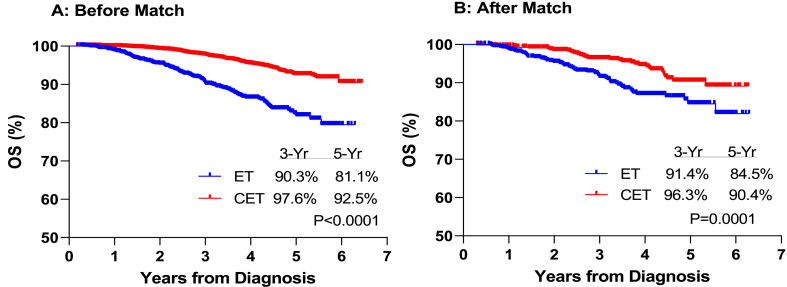
Overall Survival in the ET and CET cohorts before and after PS match.

In the unmatched sample, CET (5-year OS: 92.5%) conferred patients a survival benefit over ET (5-year OS: 81.1%) (***p<0.001***). Similar results were observed within the PS-matched sample, with a 90.4% 5-year OS in the CET cohort vs. 84.5% in the ET cohort (***p=0.001***) (Fig. 4). Results were consistent with previous survival analysis on the overall cohorts that showed survival benefit with the use of CET as compared to the other treatment modalities.

### 3.4 Survival analysis comparing the ET and CET cohorts

Looking at the 3-year OS in the group who received Anti-Her2 therapy (N=4946), the CET cohort shows a higher OS of 97.9% as compared to 89.9% in the ET cohort. Similarly, OS with CET was higher than with ET: 95.8% vs. 90.4% in those who did not receive Anti-Her2 therapy (N=1646). Examining the factors predicting the 3-year OS in the sample of patients diagnosed between 2013 and 2016 in a multivariable model stratifying for the use of Anti-Her2 therapy, treatment regimen was an independent predictor of survival: as compared to the cohort who received CET, ET was associated with lower OS in both the group who received Anti-Her2 therapy (HR: 2.56, 95% CI: 1.60,4.12, ***p<0.001***) and the group who didn't receive it (HR: 1.84, 95% CI: 1.21–2.78, ***p=0.004***). The association between treatment regimen and survival was independent of patient age, administration of radiation, tumor stage, patient comorbidity score, ILC histology and private insurance coverage. Additionally, ILC tumor histology was not significantly associated with survival: as compared to classical ILC tumor histology, mixed ILC tumor histology predicted similar survival in both the group who received Anti-Her2 therapy (HR: 0.84, 95% CI: 0.62–1.15, p=0.28) and the group who didn't receive Anti-Her2 therapy (HR: 1.03, 95% CI: 0.74–1.43, p=0.87) (Table 3).

**Table 3:** Kaplan-Meier and Cox Multivariable Survival Analyses in Anti-Her2 and no Anti-Her2 strata (2013–2016).

	Anti-Her2 Therapy			No Anti-Her2 Therapy		
	Kaplan-Meier	Cox Multivariate		Kaplan-Meier	Cox Multivariate	
	3-Year OS % (95% CI)	HR (95% CI)	*p*-value	3-Year OS % (95% CI)	HR (95% CI)	*p*-value
**Treatment cohort**						
CET	97.9 (97.4,98.4)	–	–	95.8 (94.1,97.4)	–	–
ET	89.9 (85.8,94.0)	2.56 (1.60,4.12)	***<0.001***	90.4 (88.3,92.4)	1.84 (1.21,2.78)	***0.004***
**Age category**						
<55	98.8 (98.2,99.4)	–	–	97.2 (95.4,99.0)	–	–
55–64	97.5 (96.5,98.4)	1.87 (1.19,2.92)	***0.006***	96.2 (94.1,98.3)	1.05 (0.50,2.20)	0.89
65–74	97.0 (95.8,98.1)	1.51 (0.91,2.52)	0.11	94.9 (92.7,97.1)	1.37 (0.68,2.79)	0.38
≥75	93.3 (90.6,96.1)	2.31 (1.29,4.12)	***0.005***	82.6 (78.6,86.6)	3.04 (1.51,6.11)	***0.002***
**Radiation therapy**						
Yes	97.8 (97.2,98.4)	–	–	89.4 (87.0,91.9)	–	–
No	97.0 (96.0,97.9)	1.44 (1.03,2.00)	0.031	95.1 (93.5,96.7)	1.70 (1.19,2.41)	***0.003***
**Tumor stage**						
Stage I	98.7 (98.1,99.3)	–	–	95.6 (94.1,97.0)	–	–
Stage II	97.7 (97.0,98.5)	2.07 (1.38,3.13)	***<0.001***	91.2 (88.5,93.9)	2.19 (1.49,3.23)	***<0.001***
Stage III	93.7 (91.8,95.7)	5.34 (3.49,8.18)	***<0.001***	80.8 (74.6,87.1)	7.92 (4.98,12.60)	***<0.001***
**Charlson/Deyo score**						
0	97.9 (97.4,98.4)	–		93.9 (92.4,95.3)	–	–
≥1	95.1 (93.3,96.9)	1.63 (1.14,2.31)	***0.007***	87.6 (83.7,91.4)	1.18 (0.82,1.68)	0.37
**Tumor histology**						
Classical type	97.0 (96.1,98.0)	–		91.5 (89.3,93.8)	–	–
Mixed histology	97.8 (97.2,98.4)	0.84 (0.62,1.15)	0.28	93.3 (91.6,95.1)	1.03 (0.74,1.43)	0.87
**Private Insurance**						
Yes	98.5 (98.0,99.1)	–		97.2 (95.9,98.5)	–	–
No	96.0 (95.0,97.0)	1.89 (1.27,2.81)	***0.002***	89.0 (86.7,91.3)	1.97 (1.17,3.32)	***0.011***

**Fig. 5:**
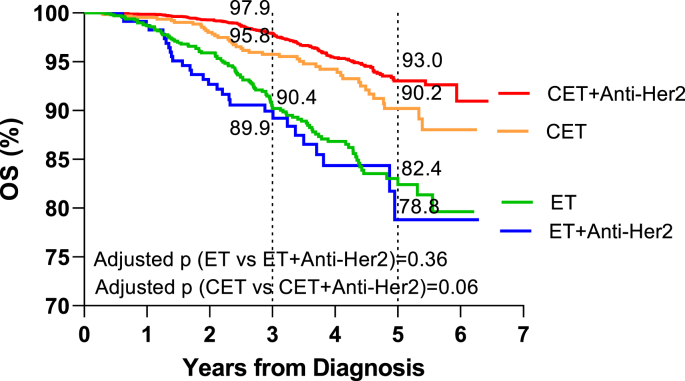
Survival Trends in ET and CET cohorts stratifying for Anti-Her2 use.

KM modeling was used to analyze OS in the CET and ET cohorts (2013–2016) stratifying for Anti-Her2 administration, adjusting for patient age, comorbidity score, insurance coverage, tumor stage, ILC histology, and RT use. 5-year OS was higher in the CET as compared to the ET cohort, regardless of Anti-Her2 administration. 5-year OS was the highest in the CET cohort who received Anti-Her2 (93.0%); it was significantly higher than OS in the CET cohort who did not receive Anti-Her2 (90.2%) (***p=0.06***). 5-year OS in the ET cohort was similar when stratifying for Anti-Her2 therapy (82.4% vs. 78.8%, p = 0.36) (Fig. 5).

## 4 Discussion

This retrospective analysis based on the large national clinical database identified trends in management and survival outcomes of chemotherapy, endocrine therapy or combined chemo-endocrine therapy in surgically resected HR+/HER2+ILC, taking into account the use of Anti-Her2 therapy. The incorporation of targeted Anti-HER2 therapy into standard systemic therapy significantly improved OS, across all study groups. The group who received combined chemo-endocrine therapy in addition to Anti-Her2 therapy had the best outcome with the highest 5-year OS, as compared to CET cohort with no concurrent Anti-Her2 therapy or ET cohort regardless of Anti-Her2 therapy, after adjusting for several variables including comorbidity and stage. Of interest, our data also suggests that combined chemo-endocrine therapy provides superior survival outcomes as compared to endocrine therapy alone in this subtype of ILC, regardless of Anti-Her2 therapy usage. The study also highlighted a significant downward trend in combined chemo-endocrine therapy usage in favor of endocrine therapy alone, in those who did not receive Anti-Her2 therapy after 2013, which likely reflects that these patients were not candidates for chemotherapy and/or Anti-Her2 targeted therapies. Newer regimens like Taxol-Herceptin that omit anthracyclines may allow for optimization of CET, as it has clear survival benefit over ET alone in node-positive ILC [17].

HR expression and HER2 amplification are major predictors of prognosis and treatment outcomes in BC [18]. Indeed, BC is classified according to the presence or absence of these receptors on the tumor cell surface, as they account for different tumorigenesis and consequent biological behavior of the cancer cells [19]. Therapeutic options thus largely depend on the receptor expression profile of BC with treatment regimens specifically targeting the various molecular subtypes [13]. Endocrine therapy halts the interaction between ER and/or PR and their ligands, and remains an essential component of the standard of care for all HR + BC since the 1970's [20]. Conversely, anti-HER2 therapies, including the monoclonal antibodies Trastuzumab or Pertuzumab have become a preordained therapeutic route in HER2+ disease [21]. In a pooled analysis of data from two phase III randomized controlled trials (RCT) in 2011, adding Anti-Her2 therapy to standard neo-adjuvant chemotherapy resulted in a 2.02 fold increase in pathologic complete response (pCR) as compared to standard chemotherapy alone (p = 0.0002), which was accompanied by a 0.67 fold decrease in relapse rate [22]. Interestingly, most of these earlier studies did not take into account cell tumor morphology and histological subtypes of breast cancer. Instead, results are generalized among the different histological subtypes and are widely based on investigations involving a majority of patients diagnosed with IDC, as the most common BC subtype. Despite emerging unique clinic-pathological and genomic features, studies focusing on ILC remain scarce, but data reporting different benefit from chemotherapy and endocrine therapy has started to emerge [[23], [24], [25], [26]].

In this retrospective analysis, we leveraged the large national clinical database to compare the management and survival outcomes of the various therapeutic regimens in early stage HR+/HER2+ILC over the years. Exploration of trends of treatment administration revealed a divergent pattern in the use of systemic therapy after introduction of Anti-Her2 therapy in 2013: while the group who received Anti-Her2 therapy after 2013 continued to be treated with systemic therapy similarly to the years before, the group of patients who did not receive Anti-Her2 therapy also received significantly more ET and less chemotherapy, i.e less CET administration. Overall, patients who did not receive Anti-Her2 therapy after 2013 were older, with more co-morbidities and lower cancer stage, and had less private insurance coverage. Historically, treatment of this same patient population with chemotherapy is complicated by concerns about comorbidities, expected tolerance, and impact on functional status [27]. Our results reveal that factors and concerns impacting the use Anti-Her2 in the clinical practice setting are similar to those impacting the use of chemotherapy. Importantly, this decline in the use of CET over time in this subset of patients who did not receive Anti-Her2 therapy goes hand-in-hand with the decline in OS observed.

Our results showed a significant survival benefit with CET as compared to ET in the overall sample of patient diagnosed between 2010 and 2013, after stratifying for Anti-Her2 therapy administration. The survival benefit of CET as compared to ET remained significant after correcting for important Anti-Her2 therapy variables that affect survival through PS matching. Tumor stage is a well-established independent predictor of survival, with more advanced stages being associated with worse survival outcomes. More advanced tumor stages were associated with administration of CET over ET, thus it was crucial to adjust for all these characteristics for proper characterization of the survival outcomes of each regimen. Our findings concerning the benefit of CET over ET contrast previous findings concerning the inconclusive benefit of chemotherapy in ILC [28], without proper categorization by Her2 status. Therefore, these study concludes that chemotherapy does provide benefit in OS in patients with HR+/HER2+ ILC.

Additionally, ILC histology did not appear to be a significant independent prognostic factor for OS. Our results from multivariable Cox proportional hazard model showed that classical ILC and mixed ILC histology were associated with similar survival outcomes. This contrasts previous data on how mixed ILC histology shows more aggressive clinical behavior than the classical ILC type and is associated with dismal prognosis [12,[29], [30], [31], [32]]. These studies were however limited by a small number of patients, a limitation that our analysis from a national registry overcomes.

This analysis is powered by a national cancer registry that is able to survey treatment administration nationwide. Limitations of our study do however include those that come with the retrospective nature of our design. While the NCDB gives information on the treatment adopted, it does not provide us with details concerning the exact chemotherapy agents administered and the regimens followed. Future research exploring the various chemotherapy and endocrine therapy treatment regimen combinations for optimal survival in HR+/HER2+ ILC need to be explored within prospective clinical trials. In addition, the survival information in the NCDB is limited to OS, and lacks information on post neo-adjuvant chemotherapy pCR, event-free survival or disease recurrence. Finally, the NCDB does not provide information on the treatment regimen received, which is important in the setting of the BIG 1–98 trial showing superior results with Letrozole ET as compared to Tamoxifen [14].

## 5 Conclusion

Our findings highlight the survival benefit of chemotherapy in HR+/HER2+ ILC, contrasting previous data on the inconclusive benefit of chemotherapy in this specific patient population. Chemotherapy followed by endocrine therapy and Anti-Her2 therapy was shown to be the most effective treatment modality in HR+/HER2+ ILC, especially in patients with larger and locally advanced tumors. This data calls for more research to investigate the need for implantation of combined chemo-endocrine therapy and Anti-Her2 therapy as the optimal treatment for ILC treatment. The role of adding adjuvant anti-HER2 therapy to ET in comparison to CET needs to be explored in future prospective trials. Finally, given the wide variability of ILC subtypes on the molecular and pathological level, the potential role of precision medicine in HR+/HER2+ ILC needs to be further investigated in the therapeutic setting.

## Funding

None of the authors have any funding to declare.

## Availability of data and material

The data that support the findings of this study are available from the American College of Surgeons/American Cancer Society but restrictions apply to the availability of these data, which were used under license for the current study, and so are not publicly available. Data are however available from the authors upon reasonable request and with permission of the American College of Surgeons/American Cancer Society.

## Code availability

Not applicable.

## Authors' contributions

M.Y., N.B. and B.D. are major contributors in study design, conduction of statistics and data interpretation, and manuscript writing. Z.N. participated in study design, interpretation of the data, and writing the manuscript. H.L. participated in the conduction of statistics. I.J., C.R., M.B.Z., D.S. and E.S. participated in writing and editing. All authors read and approved the final manuscript.

## Additional declarations for articles in life science journals that report the results of studies involving humans and/or animals

Not applicable.

## Ethics approval

Ethical approval was obtained from the Cleveland Clinic Institutional Review Board (IRB) before conducting this study. All patient data were strictly de-identified and provided, with approval, from the American College of Surgeons as part of the National Cancer Database.

## Consent to participate

Ethical approval was obtained from the Cleveland Clinic Institutional Review Board (IRB) prior to conducting this study. All patient data were strictly de-identified and provided, with approval, from the American College of Surgeons as part of the National Cancer Database.

## Consent for publication

No individual person's data were included; all data is reported in an aggregated manner.

## Declaration of competing interest

The authors declare that they have no conflict of interest. The authors declare that they have no competing interests.
